# Supplementary material for: A clinically integrated, frameless human Neuropixels workflow
Source: medRxiv. 2026 May 18:2026.05.07.26351853. Preprint. [Version 1] doi: 10.64898/2026.05.07.26351853 (PMC13228770; doi:10.64898/2026.05.07.26351853)
Supplement: Supplementary file 1 [file NIHPP2026.05.07.26351853v1-supplement-1.pdf]

medRxiv preprint doi: https://doi.org/10.64898/2026.05.07.26351853; this version posted May 18, 2026. The copyright holder for this preprint (which was not certified by peer review) is the author/funder, who has granted medRxiv a license to display the preprint in perpetuity.
It is made available under a CC-BY 4.0 International license .

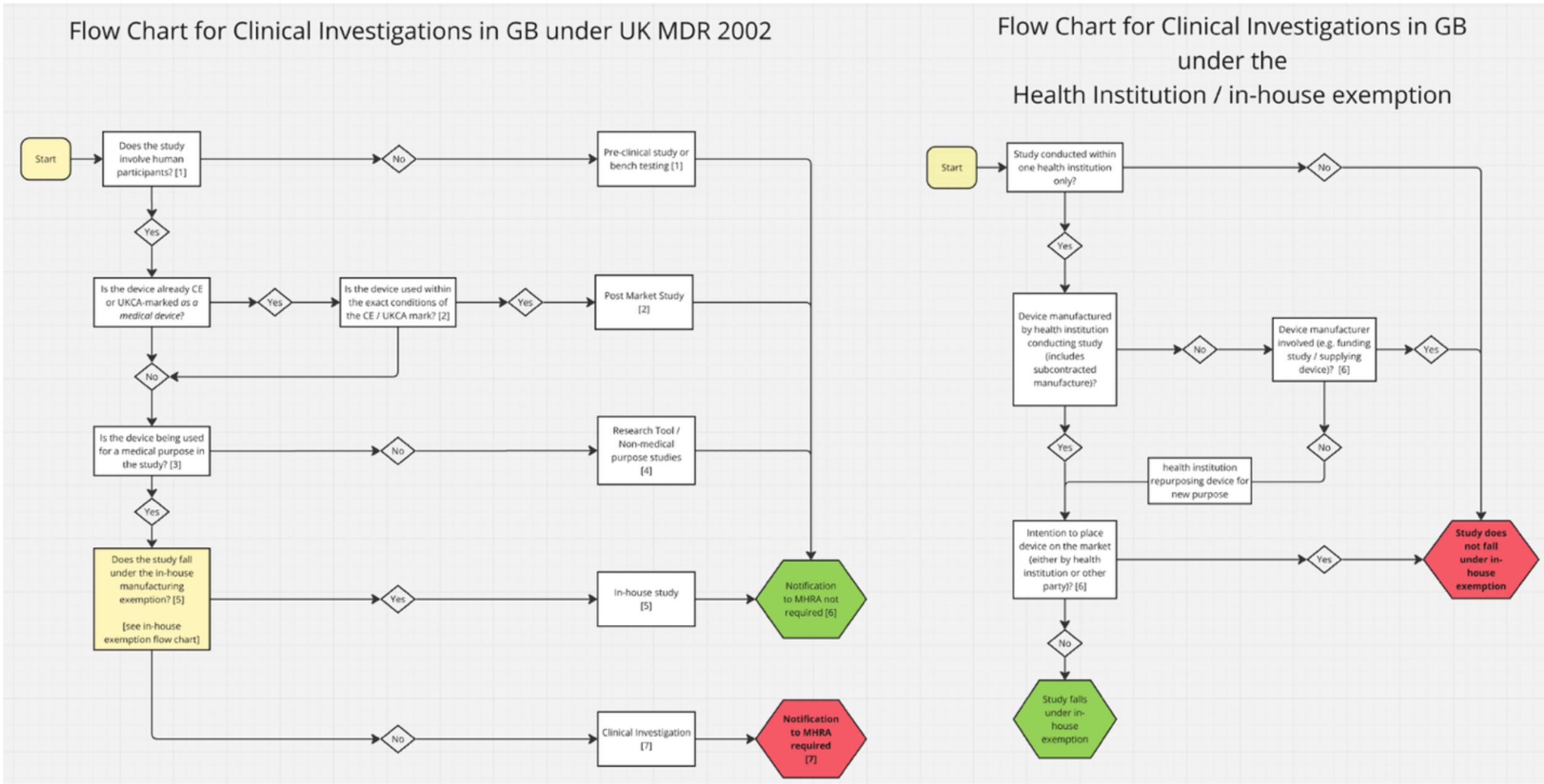

Supplementary Figure 1. Flowchart for regulatory decision making. Medicines and Healthcare products Regulatory Agency online Flow Chart for Clinical Investigations under UK Medical Device Regulations (https://assets.publishing.service.gov.uk/media/69247b5dba812a67cb6a569d/MHRA_Flow_Chart_for_Clinical_Investigations_Under_UK_MDR_Version3.png; https://www.gov.uk/guidance/notify-mhra-about-a-clinical-investigation-for-a-medical-device; accessed 01 April 2026).

medRxiv preprint doi: https://doi.org/10.64898/2026.05.07.26351853; this version posted May 18, 2026. The copyright holder for this preprint (which was not certified by peer review) is the author/funder, who has granted medRxiv a license to display the preprint in perpetuity.
It is made available under a CC-BY 4.0 International license .

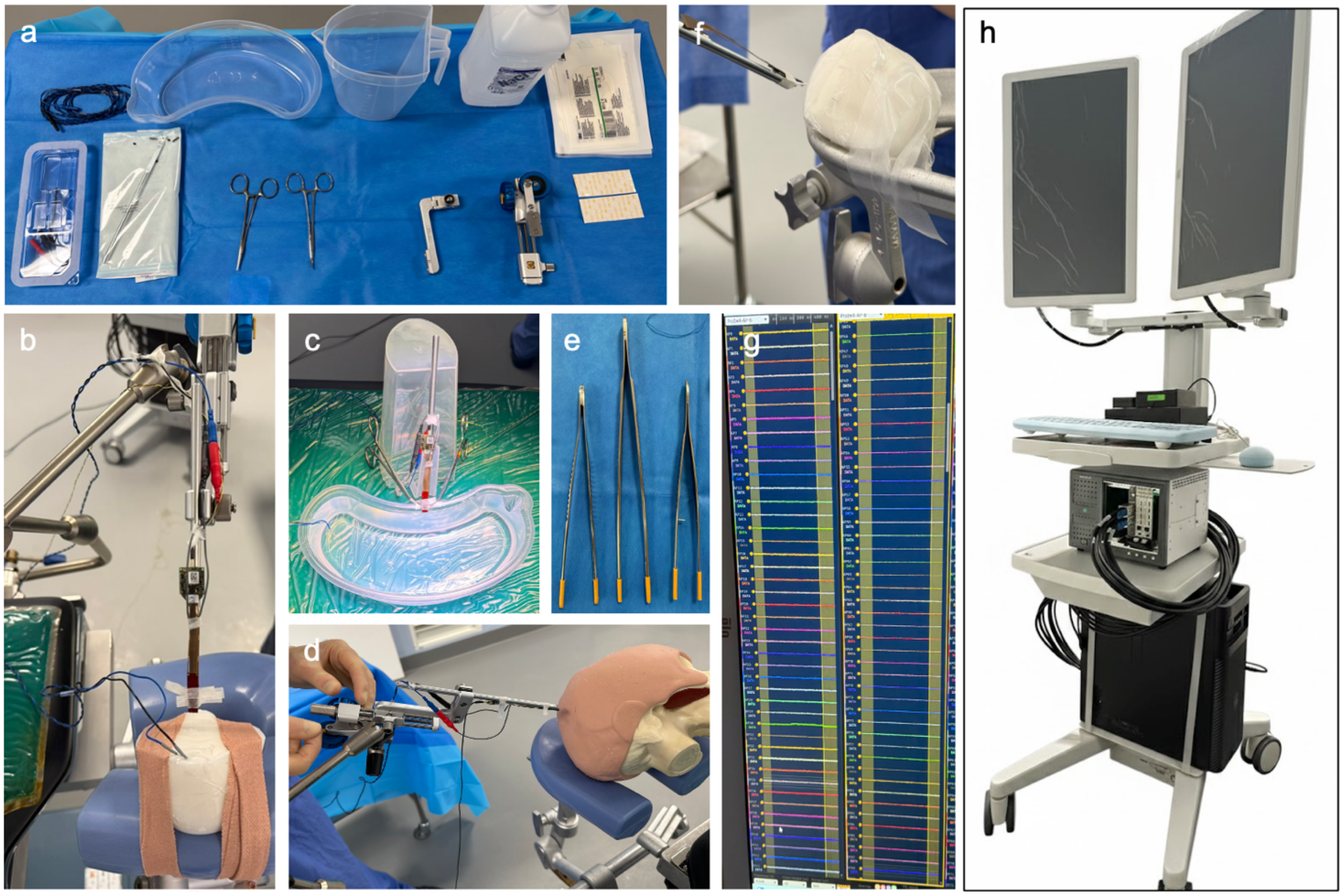

Supplementary Figure 2. Pre-clinical simulation, electrical de-noising and fracture retrieval. (a) Sterile equipment setup as on day of surgery. Equipment left to right: sterilised Neuropixels in blister pack, subdermal needles for grounding, rod holder, kidney dish for sterile saline, jug, mosquito clips, micromanipulator with attachment, steristrips. **(b)** Simulation of pial penetration using bespoke brain phantom. Karl Storz endoscope holder attaches the micromanipulator to the operating table and provides multiple degrees of freedom. **(c)** Baseline electrical noise testing on operating table with complete circuit. Theatre lights turned to maximum brightness and shone directly onto Neuropixels to assess noise. **(d)** Simulated inserted with skull phantom for ventriculoperitoneal shunt surgery with frameless technique and horseshoe headrest. **(e)** Toothed forceps protected with shunt tubing for shank fracture retrieval to prevent shank shattering. **(f)** Verification of shank retrieval without shattering using protected tooth forceps. **(g)** Example background noise from operating theatre on OpenPhys.

medRxiv preprint doi: https://doi.org/10.64898/2026.05.07.26351853; this version posted May 18, 2026. The copyright holder for this preprint (which was not certified by peer review) is the author/funder, who has granted medRxiv a license to display the preprint in perpetuity.
It is made available under a CC-BY 4.0 International license .

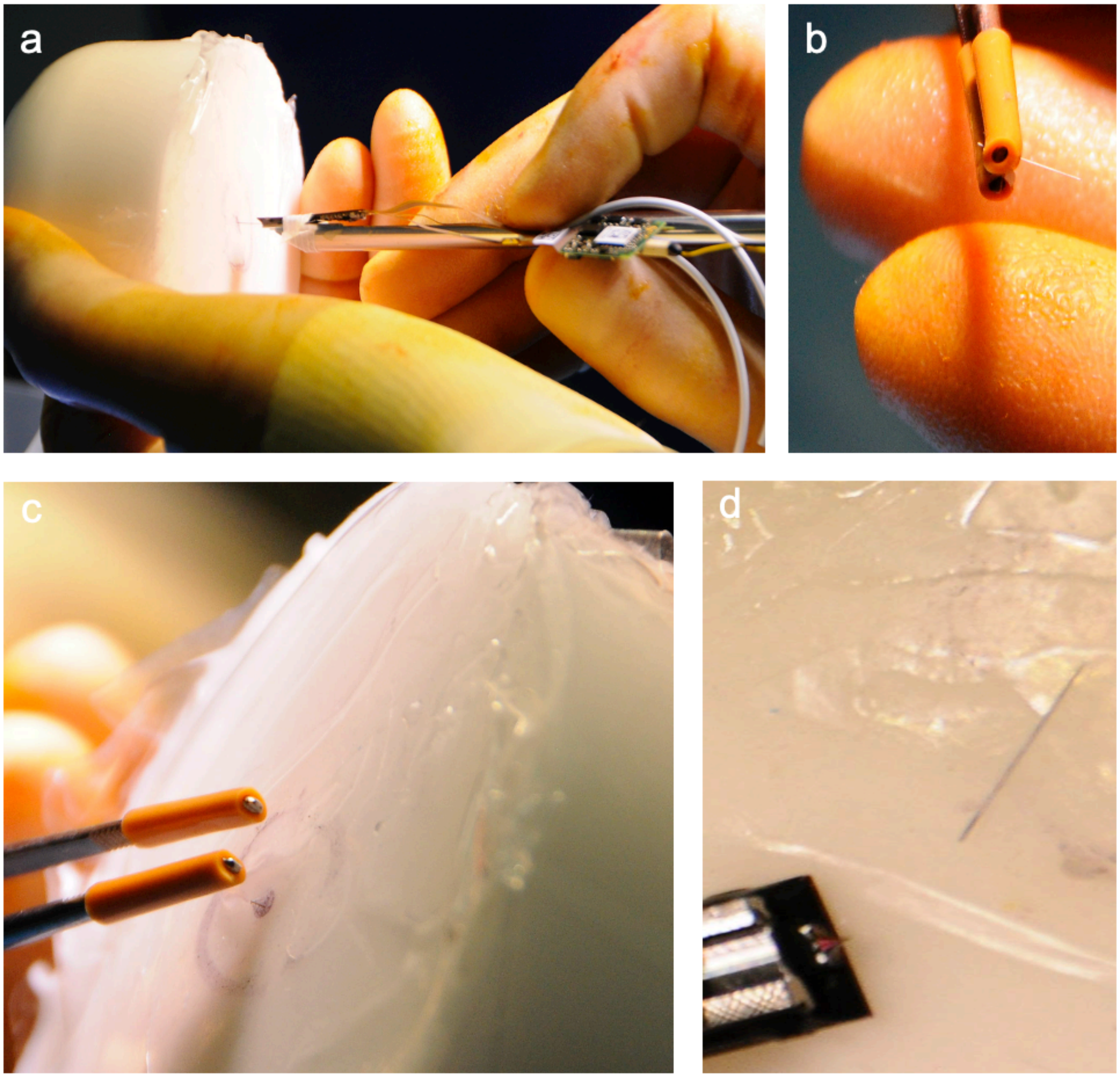

Supplementary Figure 3. Shank fracture and successful retrieval during simulation. (a) Simulating mechanical failure mode during insertion resulting in shank fracture in brain phantom (see Methods). **(b)** Using shunt tubing protected tooth forceps to prevent shank shattering, shank is removed en bloc successfully. **(c)** Difficult shank fracture retrieval with majority of shank in the "cortex". Success demonstrated in previous panel. **(d)** Confirmation of mechanical fracture occurs at proximal end of shank in agreement with literature: Paulk et al 2022, Chung et al 2022, Chung et al 2025.

medRxiv preprint doi: https://doi.org/10.64898/2026.05.07.26351853; this version posted May 18, 2026. The copyright holder for this preprint (which was not certified by peer review) is the author/funder, who has granted medRxiv a license to display the preprint in perpetuity.
It is made available under a CC-BY 4.0 International license .

Appendix 1. Pre-surgery checklist

UCL COMPREHENSIVE CLINICAL TRIALS UNIT

THE FRANCIS CRICK INSTITUTE

SUNAN

3 SUNAN Pre-surgery

Participant ID	Partial DOB:
S U _ _ _	M M M - Y Y Y Y

DATE OF VISIT	D D – M M M – Y Y Y Y

Checklist

		YES (✓)	NO (✓)
**1.**	Ongoing consent from participant		
**2.**	Inclusion and exclusion criteria met and participant eligible for SUNAN Study?		
**3.**	Confirm consent form for SUNAN study signed by participant?		
**4.**	Confirmation of Pregnancy/breastfeeding status for Women of Childbearing Potential? ☐ N/A (Male, or >65years female)		
**4a.**	Urine or Blood Test? ____________________ ☐ N/A		
**4b.**	Pregnancy test Negative? ☐ N/A		
**5.**	Confirmation by consultant anaesthetist the ASA grade of participant?		
**6.**	Confirmation by consultant neurosurgeon suitable for study?		
**7.**	Confirmation by consultant anaesthetist suitable for study?		
**8.**	Will the participant be awake or asleep?	**Awake**	**Asleep**
**9.**	If awake, have the behavioural tasks been agreed?		
**9a.**	What tasks have been agreed?		

SUNAN/ Eligibility & Randomisation CRF / Version 1.0 / 19Jun2025 Page 1 of 3
UCL Comprehensive Clinical Trials Unit, 90 High Holborn, 2nd Floor, London, WC1V 6LJ / Email: CCTU.SUNAN@ucl.ac.uk

medRxiv preprint doi: https://doi.org/10.64898/2026.05.07.26351853; this version posted May 18, 2026. The copyright holder for this preprint (which was not certified by peer review) is the author/funder, who has granted medRxiv a license to display the preprint in perpetuity.
It is made available under a CC-BY 4.0 International license .

Participant ID: S U _ _ _ | Partial DOB: M M M - Y Y Y Y

	if yes to the domain, please specify each test to be performed		
	Motor Tasks:		
	Sensory Tasks:		
	Speech Tasks:		
	Cognition Tasks:		
	Visual Tasks:		
**10.**	Neurophysiological recording process and roles confirmed with surgical and study team?		
**11.**	Did the patient consent to CSF sub-study and has continued consent?		
**12.**	Did the patient consent to Tissue Biopsy sub study and has continued consent?		
**13.**	If consented to sub-study, discussion of sample management and transfer? N/A ☐		
**14.**	Time of completion:		

Form Completed by:

Signature:_________________________

Date:

914

v	Case	E2: Duration, min		E3: Data, units Yield	Failure mode		Root cause	Mitigation	Iteration	Verification
		Research	Record							
**0.1**	SU000	Pre-clinical theatre simulation, fracture simulation, electrical de-noising								Baseline pipeline: 1.0-S probe, PXIe acquisition system, fracture SOP
**1.0**	SU001	28	7	25	Major	NA	NA	NA	NA	Simulated workflow and timing validated in real life, proof of units
					Minor				2 x large trolleys for more space during preparation	
**1.1**	SU002	20	-	0	Major	Noise	Neuronavigation (EM)	First case with EM, iterated workflow for isolation prior to insertion, post-operative testing confirming EM source of noise	Unplug EM cable prior to Neuropixels insertion	Noise reduction in saline circuit set up
					Minor					
**1.2**	SU003	20	-	0	Major	Noise	Neuronavigation (EM instrument port)	Full EM instrument port isolation protocol (disconnecting green cable) + confirmed registration remained when plugged back in	Further refined de-noising protocol and "Critical" communication section in workflow prior to insertion	No further noise disruption from neuronavigation
					Minor	Time			Added accessible stopwatch to cart	Improved time management
**1.3**	SU005	27	10	70	Major	Stability	Setup stability	Changed clamp holder	Improve holder micromanipulator attachment	Better unit yield by reduced mechanical drift
					Minor	Noise	Electrocautery, noisy first 2 minutes whilst debugging	Turn off and unplug	Formally added to de-noising checklist	No further noise from electrocautery
						Insertion	Slow insertion speed based on literature	Insertion using micromanipulator over 2-3m	Faster insertion speed	Fast insertion speed ~3-4s for SU007
**1.4**	SU006	29	2	0	Major	Technical	PXIe instability	Pivot to imec Onebox	Onebox as primary acquisition device, computer optimisations	System boot-up time improved from 3-5m to <30s
					Minor	Hardware	Headstage not read by PXIe	Opened new items, had to leave theatre to get item,	Bring 3 x sterile equipment to theatre	Improved workflow
**2.0**	SU007	24	15	146	Major		NA	Stabilised pipeline		High-fidelity unit yield
					Minor					

Supplementary Table 1. Iterative technical and procedural refinements during IDEAL Stage 2a Development.

Prospective documentation of iterative pipeline maturation across consecutive surgical cases using the IDEAL Stage 2a Development framework. Each version reflects modifications introduced in response to observed failure modes with key Endpoints (E) of Duration and Data. Notifiable Safety Events were not included in this table as there were none. Failure mode was categorised as Major or Minor depending on impact on E2 and E3. Mitigation strategies were implemented and validated in subsequent cases. Recording duration and data yield improved iteratively, culminating in a stabilised pipeline (v2.0) achieving 146 putative units during the full 15-minute recording window. These iterative refinements informed the development of standardised workflows, intraoperative de-noising protocols, and integration of hospital-compatible acquisition hardware. Further pre-operative and post-operative iterations relating to recruitment, consent, data management, and general study iterations are not considered in this table. v: Version of pipeline; E2: Endpoint 2; E3: Endpoint; EM: electromagnetic neuronavigation
